# Association between vitamin D levels and depression among type 2 diabetes mellitus patients in the Southern Region of Malaysia

**DOI:** 10.11604/pamj.2026.53.116.47001

**Published:** 2026-03-09

**Authors:** Norizzati Amsah, Zaleha Md Isa, Norfazilah Ahmad, Zaid Kassim, Siti Nor Athirah Mohamad

**Affiliations:** 1 Family Health Research Centre, Institute for Public Health, National Institutes of Health, Setia Alam, Malaysia,; 2 Department of Public Health Medicine, Universiti Kebangsaan Malaysia, Bandar Tun Razak, Malaysia,; 3 Tangkak District Health Office, Johor State Health Department, Tangkak, Johor, Malaysia,; 4 Segamat District Health Office, Johor State Health Department, Segamat, Johor, Malaysia

**Keywords:** Vitamin D, depression, diabetes, mental health, type 2 diabetes mellitus

## Abstract

**Introduction:**

depression is a significant public health concern among patients with type 2 diabetes mellitus (T2DM) worldwide. Vitamin D, essential for brain health, plays a role in neuroinflammation and neurotransmitter synthesis, processes linked to depression. However, few studies have explored the association between vitamin D levels and depression in T2DM patients.

**Methods:**

this cross-sectional study investigated the relationship between serum vitamin D levels and depression among 330 T2DM patients in Johor, Malaysia. Serum 25-hydroxyvitamin D levels were measured using ultra-performance liquid chromatography (UPLC) and categorized as deficient (<30 nmol/L), insufficient (30??50 nmol/L), or sufficient (>50 nmol/L). Depressive symptoms were assessed using the Patient Health Questionnaire-9 (PHQ-9).

**Results:**

the prevalence of depression was 35.5%. Multiple logistic regression analysis revealed that depression was significantly associated with older age (>60 years) (adjusted OR 2.04, 95% CI: 1.23-3.38), vitamin D deficiency (adjusted OR 4.24, 95% CI: 1.99-8.03), vitamin D insufficiency (adjusted OR 1.62, 95% CI: 1.19-2.71), and the use of both oral and injection medications (adjusted OR 1.86, 95% CI: 1.05-3.31).

**Conclusion:**

ensuring adequate vitamin D levels, particularly in elderly T2DM patients, may reduce the risk of depression, emphasizing the importance of integrated and holistic management strategies. Keywords: Vitamin D, depression, diabetes, mental health, type 2 diabetes mellitus.

## Introduction

Type 2 diabetes mellitus (T2DM) is a chronic metabolic disorder characterized by insulin resistance and impaired glucose metabolism that affects millions of people worldwide. In addition to its established associations with cardiovascular disease and other physical complications, T2DM is increasingly recognized for its relationship with mental health, particularly depression [1]. Depression is a common comorbidity of T2DM, leading to reduced quality of life, poor glycaemic control, and increased mortality [2]. Depression is more prevalent among T2DM patients than in the general population, likely because of the disease´s chronic nature, such as metabolic dysregulation, psychological stress, and lifestyle restrictions [3]. Previous research suggests that T2DM patients with depression have poor glycaemic control, an increased risk of complications, and further reductions in quality of life [4]. Several factors influencing mental health in T2DM, particularly vitamin D level, have attracted significant attention [5,6]. Vitamin D, commonly known for its role in bone health and immune function, is now gaining attention for its potential impact on mental health [7].

Previous research indicates that vitamin D deficiency is more prevalent among patients with T2DM than in the general population [8]. Those with T2DM and depression usually have low vitamin D levels [9]. Past literature suggests that low vitamin D levels are associated with a greater risk of depression due to their impact on brain function, inflammation, and neurotransmitter regulation [10,11]. Notably, vitamin D plays a role in synthesizing serotonin, which is critical in mood and emotional regulation [12]. While numerous past studies have explored these relationships among the general population, research on the impact of vitamin D on mental health in Malaysian T2DM patients remains limited. Given the complex interplay between physical health, clinical factors, and mental well-being in T2DM patients, understanding the role of vitamin D in depression is essential. This study hypothesizes that lower vitamin D levels are significantly associated with an increased risk of depression among individuals with type 2 diabetes mellitus (T2DM). It is proposed that vitamin D deficiency, potentially due to limited sun exposure, dietary intake, or metabolic factors, contributes to poorer mental health outcomes in this population. Therefore, this study aims to identify the association between vitamin D levels and depression among T2DM patients in Johor, Malaysia, providing insights that could inform public health interventions and individualized management strategies for this at-risk population.

## Methods

**Study design:** this cross-sectional study included 330 T2DM patients across seven healthcare facilities in Johor, which is a southern part of Peninsular Malaysia. The predominant ethnic group is Malay, followed by Chinese and Indian communities, reflecting the ethnic diversity found in Malaysia [13].

**Figure 1:**
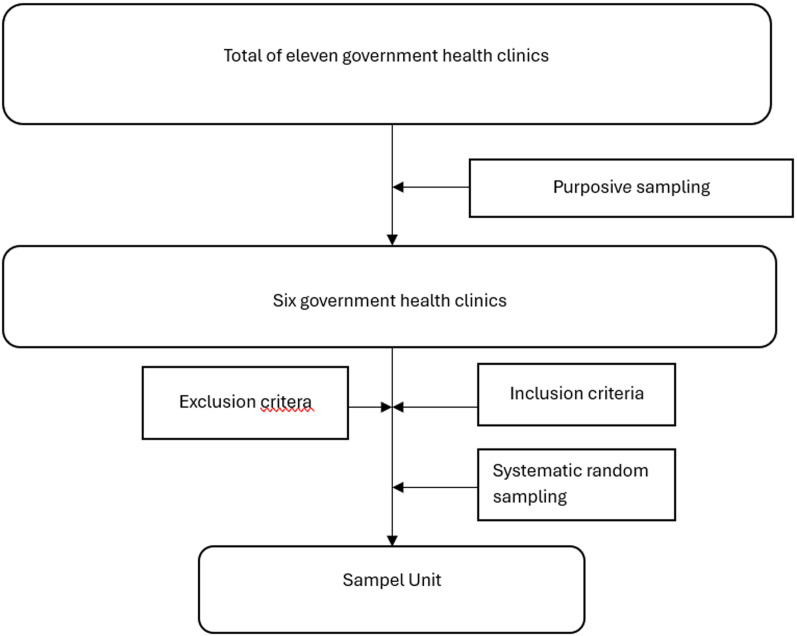
flow for selection of respondents

**Study setting:** six clinics were selected based on the highest number of patients with T2DM from eleven health clinics. The flow of the study is explained in Figure 1.

**Participants:** the inclusion criteria encompassed patients with T2DM lasting six months or more who were at least 18 years old. Those who were pregnant were excluded from the study. Respondents were then selected using a systematic random sampling method based on the appointment lists from each selected clinic. These lists were screened according to predetermined inclusion and exclusion criteria. The appointment lists of patients scheduled to attend the selected clinics were obtained each week. Respondents were chosen using systematic random sampling. For systematic random sampling, the total population of T2DM patients at this study location is approximately 9,000. Dividing this by the sample size of 330 yields an interval value of three. Respondents are then selected at every third interval until the required sample size is reached.

**Variables:** depression was assessed using the Patient Health Questionnaire-9 (PHQ-9), a validated nine-item tool for screening depression [14]. The PHQ-9 assesses the frequency of depressive symptoms over the past two weeks based on Diagnostic and Statistical Manual of Mental Disorders-IV criteria, with scores ranging from 0 (none) to 3 (nearly every day) [14]. In this study, a PHQ-9 score of more than 4 was classified as indicative of depression (Figure 1).

**Data sources/measurement:** the respondents provided demographic and socioeconomic information, including age, gender, race, total household income, working status, education level, and marital status. Clinical characteristics included body mass index, HbA1c level, and types of medication. Gender, race, total household income, working status, education level, and marital status were collected face-to-face. Clinical characteristics included body mass index, HbA1c level, and types of medication collected face-to-face and referred to health records. A blood sample of 5 mL was taken from each respondent for serum vitamin D level. Respondents were asked to fast for at least 8 hours before blood withdrawal. Serum vitamin D levels were measured using the Ultra-Performance Liquid Chromatography (UPLC) method in the UKM Medical Molecular Biology Institute (UMBI), Malaysia. This method is a method known for its sensitivity and specificity in determining vitamin D concentrations [15]. The vitamin D levels were categorized as follows: <30 nmol/L (deficient), 30-50 nmol/L (insufficient), and >50 nmol/L (sufficient) [15].

**Bias:** for selection bias, the appointment lists of patients scheduled to attend the selected clinics were obtained each week. Respondents were chosen using systematic random sampling. A p-value of less than 0.05 was used to determine the statistical significance of publication bias. To mitigate bias from missing data, multiple imputations were applied instead of excluding incomplete cases. Subgroup analyses were carried out across different age groups to identify potential biases within these specific populations. Validated questionnaires were employed to minimize measurement errors and related biases. Statistical analyses were conducted using SPSS version 27, ensuring consistency and reliability in computations.

**Sample size determination:** the sample size was determined using the Kish formula (1965) and two proportions. Based on the prevalence of depression, 26% by Miskan and Ambiga, with an α of 0.05 and a power of 0.80, the required sample size was calculated as 296 [16]. Adding 10% to account for potential dropouts resulted in a target sample size of 329.

**Quantitative variables:** continuous variables were categorized based on the guidelines and definitions: for age group (years), less than 60 and more than 60; HbA1c level, less than 6.5% and below, and more than 6.5%. Vitamin D level, less than 30 nmol/L (deficiency), 30 to 50 nmol/L (insufficiency), and 50 nmol/L (sufficient). For income, RM 4,859 and below, and RM 4,860 until RM 10,679.

**Data analysis:** data were analyzed using SPSS version 27 (IBM SPSS, New York, USA). Descriptive statistics summarized the sociodemographic and clinical characteristics. Simple and multivariate logistic regression were used to examine factors associated with depression, adjusting for potential confounders such as gender, race, total household income, employment status, education level, and marital status. Clinical characteristics included body mass index (BMI), HbA1c level, and types of medication. Factors associated with depression were analysed using simple and multivariate logistic regression analysis. All statistical analyses were carried out at a 95% confidence interval or p-value < 0.05.

**Ethical consideration:** this study was registered with the National Medical Research (NMRR) and the Medical Research and Ethics Committee (MREC), Ministry of Health, with the registration number NMRR ID (NMRR-22-01212-XHN (IIR)). Ethics approval was also obtained from the Ethics and Research Committee of the National University of Malaysia (UKM) [FF-2022-252].

## Results

**Total respondents:** total respondents in this study is 330 T2DM patients, and the response rate was 100%.

**Sociodemographic characteristics of T2DM patients:** the descriptive data of the respondents were shown in Table 1. The mean age of the respondents was 62.49 years (SD: 10.64). A total of approximately 65.5% of respondents are aged 60 years and above. Most respondents were Malays (54.5%), followed by Chinese (28.2%) and Indians (17.3%). A total of 62.7% of the respondents were women. For educational status, 45.5% of the respondents received primary education, followed by secondary and tertiary education. For marital status, most respondents were married (95.8%), followed by unmarried (4.2%). A total of 48.5% of the respondents were employed. Most respondents (74.5%) took oral medication, 20% took oral and insulin injections, while 5.5% took injections alone. As for weight, 14.9% of the respondents were overweight, and 49.4% were obese. The respondent´s mean HbA1c level was 7.67% (SD1.92), and 67.9% had an uncontrolled HbA1c level (>6.5%). Most respondents (94.2%) had an income of less than RM 4,860. A total of 95.8% of the respondents were married, and 4.2% were unmarried.

**Table 1:** sociodemographic and clinical characteristics of the respondents

Variables	Mean (standard deviation)	Frequency (%)
**Age (years)**	62.49(10.64)	
**Age group (years)**		
<60		114 (35.5)
≥60		216 (65.5)
**Gender**		
Men		123 (37.3)
Woman		207 (62.7)
**Race**		
Malay		180 (54.5)
Chinese		93 (28.2)
India		57 (17.3)
**Education level**		
Primary education		150 (45.5)
Secondary education		148 (44.9)
Tertiary education		32 (9.6)
**Marital status**		
Married		316 (95.8)
Not married		14 (4.2)
**Status of working**		
Working		160 (48.5)
Not working		170 (51.5)
**Income**		
RM 4,859 and below		311 (94.2)
RM 4,860 until RM 10,679		19 (5.8)
**Diabetes treatment**		
Oral		246 (74.5)
Injection		18 (5.5)
Oral and Injections		66 (20.0)
**Body mass index**		
Normal		118 (35.7)
Overweight		49 (14.9)
Obese		163 (49.4)
**HbA1c (%)**	7.67 (1.92)	
≤6.5%		106 (32.1)
>6.5%		224 (67.9)
**Vitamin D level (**nmol/L**)**	49.26 (15.21)	
< 30 (deficiency)		40 (12.1)
30 to 50 (insufficiency)		153 (46.4)
> 50 (sufficient)		137 (41.5)

**Vitamin D level among T2DM patients:** the mean vitamin D concentration among the respondents was 49.26 nmol/L (SP15.21). A total of 12.1% of the respondents were classified as vitamin D deficient, 46.4% as with vitamin D insufficiency, and 41.5% as vitamin D adequate. Overall, 58.5% of the respondents had low serum vitamin D levels.

**Depression and age distribution among T2DM patients:** a total of 117 respondents (35.5%) had a PHQ-9 score of more than 4 (Table 2). A total of 117 respondents (35.5%) had a PHQ-9 score of more than 4 (Table 2). Among the respondents with depression, 70.9% were aged 60 years and above.

**Table 2:** prevalence of depression and age distribution among respondents

Variables	Frequency (%)
**Depression**	
Yes	117 (35.5)
< 60 years old	34 (29.1)
≥ 60 years old	83 (70.9)
No	213 (64.5)

**Factors associated with depression: age, vitamin D level, and the use of both oral and injection medications:** the multivariate analysis showed significant associations between depression and several factors, including older age (≥ 60 years) (adjusted OR 2.04, 95% CI: 1.23-3.38), vitamin D deficiency (adjusted OR 4.24, 95% CI: 1.99-9.03), vitamin D insufficiency (adjusted OR 1.62, 95% CI: 1.19-2.71), and the use of both oral and injection medications (adjusted OR 1.86, 95% CI: 1.05-3.31) in Table 3, Table 4. The other confounders, such as gender, race, education level, marital status, income, BMI, and HbA1c level, are not significant.

**Table 3:** simple logistic analysis of the factors associated with depression among type 2 diabetes mellitus patients

Variables	Crude odds ratio	95%CI		P-value
	Exp (B)	Lower	Upper	
**Age**				
<60 years (reference)	1.00			
≥60 years	1.60	1.18	2.93	0.035*
**Gender**				
Male (reference)	1.00			
Female	1.13	0.63	2.02	0.687
**Race**				
Malay	0.84	0.39	2.80	0.647
Chinese	1.07	0.47	2.46	0.866
Indian (reference)	1.00			
**Education level**				
Primary education	0.95	0.46	0.54	0.920
Secondary education	0.97	0.36	2.58	0.946
Tertiary education (reference)	1.00			
**Marital status**				
Married (reference)	1.00			
Unmarried	1.24	0.34	4.59	0.748
**Status of working**				
Work (reference)	1.00			
Not working	0.96	0.62	1.49	0.867
**Income**				
RM 4,859 and below	1.44	0.55	3.76	0.456
RM 4,860 until RM 10,679 (reference)	1.00			
**Body mass index**				
Normal (reference)	1.00			
Obese	0.69	0.52	1.43	0.912
**HbA1c**				
≤6.5%(reference)	1.00			
>6.5%	1.18	0.74	1.87	0.498
**Diabetes treatment**				
Oral medication (reference)	1.00			
Injection	0.57	0.29	3.72	0.337
Oral medication and injection	1.88	1.08	3.28	0.027*
**Vitamin D levels**				
<30 nmol/L(deficiency)	5.98	1.86	6.55	0.006*
30 - 50 nmol/L(insufficiency)	3.12	1.60	6.08	0.007*
>50 nmol/(sufficient) (reference)	1.00			

**Table 4:** multivariate analysis of the factors associated with depression among type 2 diabetes mellitus patients

Variables	Adjusted odds ratio	95%CI		P-value
	Exp (B)	Lower	Upper	
**Age**				
<60 years (reference)	1.00			
≥60 years	2.04	1.23	3.38	0.006*
**Gender**				
Male (reference)	1.00			
Female	1.03	0.54	1.97	0.719
**Race**				
Malay	1.55	0.87	2.76	0.135
Chinese	0.63	0.31	1.26	0.206
Indian (reference)	1.00			
**Education level**				
Primary education	1.22	0.46	3.23	0.687
Secondary education	1.17	0.44	2.80	0.544
Tertiary education (reference)	1.00			
**Marital status**				
Married (reference)	1.00			
Unmarried	1.25	0.36	4.47	0.715
**Status of working**				
Work (reference)	1.00			
Not working	0.79	0.43	1.477	0.465
**Income**				
RM 4,859 and below	2.13	0.83	5.72	0.089
RM 4,860 until RM 10,679 (reference)	1.00			
**Body mass index**				
Normal (reference)	1.00			
Obese	1.04	0.62	1.75	0.872
**HbA1c**				
≤6.5% (reference)	1.00			
>6.5%	1.36	0.27	0.79	0.274
**Diabetes treatment**				
Oral medication (reference)	1.00			
Injection	0.64	0.19	2.09	0.456
Oral medication and injection	1.86	1.03	3.31	0.035*
**Vitamin D levels**				
<30 nmol/L (deficiency)	4.24	1.90	8.03	0.001*
30-50 nmol/L (insufficiency)	1.62	1.07	2.71	0.038*
>50 nmol/ (sufficient) (reference)	1.00			

Using *the forward LR (likelihood ratio) method*; CI: confidence interval; Hosmer and Lemeshow test, p=0.663, *Significant at p <0.05

## Discussion

**Older age higher risk of depression among T2DM:** the age group of the respondents in this study was divided at the cut-off point of 60 years on the basis of the definition of elderly in Malaysia [17]. The analysis showed that patients 60 years old and above were associated with depression. It was shown that depression is more prevalent among the T2DM patients who are 60 years and above in this study. The results of this study are consistent with a previous study in a hospital setting in Malaysia that revealed that people over the age of 50 were more likely to have high depression scores in patients with T2DM [18]. Similar trends have been observed in studies from China and Pakistan, where older age was identified as a risk factor for depression among individuals with T2DM [19,20]. Malaysia is heading toward an aging nation, and increasing life expectancy is causing more elderly people with diseases such as T2DM to suffer from depression due to complication-related diseases [21]. The high rate of depression is due to factors related to aging, such as living alone, financial problems, poor health, and cognitive dysfunction [22]. Past studies have shown that depression among elderly patients with T2DM is due to various health problems and decreased physical function, including decreased muscle mass, decreased visual and hearing levels, and bone and joint problems [23,24].

**Vitamin D level among T2DM patients:** in this study, patients with T2DM who had serum vitamin D levels less than 30 nmol/L, who were vitamin D deficient, and those who had serum vitamin D levels ranging from 30 to 50 nmol/L (vitamin D insufficiency) had a higher risk of depression [25]. Various studies have reported that low vitamin D levels are linked to various diseases, such as depression, T2DM, cardiovascular disease, and cancer [7,26,27]. Vitamin D is involved in various pathways, such as neuroimmunomodulator processes and brain development, which are linked to depression [28]. Previous studies have shown that receptors for vitamin D are present in neurons, including in the cingulate cortex and hippocampus, which are involved in depressive pathophysiology [29].

Vitamin D plays an important role in regulating insulin levels and maintaining proper glucose metabolism in patients with T2DM [27]. Type 2 diabetes mellitus patients with depression tend to have higher blood sugar levels, suggesting one contributing factor for increased stress and indirectly worsening the depression [30]. A cohort study also revealed that low vitamin D levels are one of the factors for the incidence of depressive symptoms in elderly patients [31]. After six years of follow-up, respondents with low vitamin D levels had a higher risk of developing depressive symptoms [31]. A meta-analysis of 14 studies showed lower levels of vitamin D in patients with depression compared to healthy individuals [10]. In addition, a placebo-controlled randomized clinical trial study showed that vitamin D supplementation was effective in reducing mild to moderate depression in patients with T2DM [32].

**Oral pills and injection higher risk of depression:** based on our findings, the patients who are on oral pills and injection medication appear to have a higher risk of depression compared to T2DM patients with oral pills. A systematic review involving 12 studies revealed that insulin therapy was associated with the risk of depression [33]. The treatment burden can increase stress and fatigue, contributing to an elevated risk of depressive symptoms [34]. The necessity for patients to manage multiple medications can lead to significant emotional distress. This is due to the complexity of managing multiple medications, which can make patients feel overwhelmed or unable to fully control their condition, potentially increasing their perception of illness severity [3,5]. As managing diabetes becomes more challenging, patients may experience diminished satisfaction with their overall well-being, contributing to poor mental and emotional health outcomes [3,6]. Addressing these mental health needs can be critical for improving the overall quality of life and well-being of T2DM patients who rely on both oral and injectable medications.

**Strength and limitations:** this is the first study measuring serum vitamin D levels among T2DM patients and examining the relationship between vitamin D levels and depression in Malaysia. We used biochemical assessment to measure serum vitamin D levels in this study. As this is a cross-sectional study, it can only establish associations rather than causal relationships between vitamin D levels and depression. Depressive symptoms were assessed using the Patient Health Questionnaire (PHQ-9), which is a self-reported tool. This could introduce self-reported biases.

## Conclusion

This study highlights the significant association between depression and older age, low vitamin D levels, and the use of both oral and injection medications among T2DM patients in a part of southern Malaysia. These findings underscore the importance of addressing vitamin D deficiency and insufficiency, especially in elderly T2DM patients, as a potential strategy for reducing the risk of depression. Given the high burden of depression in this population, targeted interventions to improve vitamin D status and mental health support are essential in the comprehensive management of T2DM patients. Future research should explore how these findings apply to elderly populations in different settings beyond Malaysia. Investigating the impact of sociodemographic and clinical factors on depression among older adults in diverse cultural and healthcare contexts could provide valuable insights.

### What is known about this topic

- Depression is a common comorbidity among patients with type 2 diabetes mellitus (T2DM) and is associated with poorer health outcomes;
- Vitamin D plays a crucial role in brain health, influencing neuroinflammation and neurotransmitter regulation, both of which are linked to depression;
- Higher prevalence of vitamin D deficiency and insufficiency among T2DM patients.

### What this study adds

- This study provides evidence that vitamin D deficiency and insufficiency are significantly associated with an increased risk of depression in T2DM patients;
- Older age (>60 years) and the use of both oral and injectable diabetes medications were identified as additional risk factors for depression in this population;
- The findings highlight the importance of addressing vitamin D deficiency as a potential modifiable factor in the integrated management of T2DM and mental health.
